# LMTRDA: Using logistic model tree to predict MiRNA-disease associations by fusing multi-source information of sequences and similarities

**DOI:** 10.1371/journal.pcbi.1006865

**Published:** 2019-03-27

**Authors:** Lei Wang, Zhu-Hong You, Xing Chen, Yang-Ming Li, Ya-Nan Dong, Li-Ping Li, Kai Zheng

**Affiliations:** 1 Xinjiang Technical Institute of Physics and Chemistry, Chinese Academy of Science, Urumqi, China; 2 School of Information and Control Engineering, China University of Mining and Technology, Xuzhou, China; 3 Department of Electrical Computer and Telecommunications Engineering Technology, Rochester Institute of Technology, Rochester, United States of America; 4 Xiangya School of Public Health, Central South University, Changsha, China; Ottawa University, CANADA

## Abstract

Emerging evidence has shown microRNAs (miRNAs) play an important role in human disease research. Identifying potential association among them is significant for the development of pathology, diagnose and therapy. However, only a tiny portion of all miRNA-disease pairs in the current datasets are experimentally validated. This prompts the development of high-precision computational methods to predict real interaction pairs. In this paper, we propose a new model of Logistic Model Tree for predicting miRNA-Disease Association (LMTRDA) by fusing multi-source information including miRNA sequences, miRNA functional similarity, disease semantic similarity, and known miRNA-disease associations. In particular, we introduce miRNA sequence information and extract its features using natural language processing technique for the first time in the miRNA-disease prediction model. In the cross-validation experiment, LMTRDA obtained 90.51% prediction accuracy with 92.55% sensitivity at the AUC of 90.54% on the HMDD V3.0 dataset. To further evaluate the performance of LMTRDA, we compared it with different classifier and feature descriptor models. In addition, we also validate the predictive ability of LMTRDA in human diseases including Breast Neoplasms, Breast Neoplasms and Lymphoma. As a result, 28, 27 and 26 out of the top 30 miRNAs associated with these diseases were verified by experiments in different kinds of case studies. These experimental results demonstrate that LMTRDA is a reliable model for predicting the association among miRNAs and diseases.

## Author summary

Identification of miRNA-disease associations is considered as an important step for the development of diagnose and therapy. Computational methods contribute to discovering the potential disease-related miRNAs. Based on the assumption that functionally related miRNAs tend to be involved disease, the model of LMTRDA is proposed to prioritize the underlying miRNA-disease associations by fusing multi-source information including miRNA sequences, miRNA functional similarity, disease semantic similarity, and known miRNA-disease associations. Through cross validation, the promising results demonstrated the effectiveness of the proposed model. We further implemented the case studies of three important human complex diseases including Breast Neoplasms, Breast Neoplasms and Lymphoma, 28, 27 and 26 of top-30 predicted miRNA-disease associations have been manually confirmed based on recent experimental reports. It is anticipated that LMTRDA model could prioritize the most potential miRNA-disease associations on a large scale for advancing the progress of biological experiment validation in the future, which could further contribute to the understanding of complex disease mechanisms.

## Introduction

MicroRNAs (miRNAs) are a small class of endogenous non-coding RNAs with a length of about 20–24 nucleotides [1]. They bind to the 3'-untranslated region of target miRNA through sequence-specific base pairing, resulting in cleavage or translation inhibition of target miRNA, and thereby regulating gene expression at the post-transcriptional level [2]. A growing body of research has shown that miRNA plays an important role in many biological processes, and their mutations and dysfunctions may lead to a variety of diseases [3]. Therefore, it is very important to identify the relationship among miRNAs and diseases, which has become a research hotspot in recent years.

Early studies often use biological experiments to determine the impact of a single factor on the results of the experiment and achieve higher accuracy. Lee *et al*. discovered the first miRNA in 1993, that is, the presence of Lin-4 in *C*.*elegans* [4]. Since then, many miRNAs have been discovered and identified by using different biological experimental methods, thus giving new insights into the functions and regulatory mechanisms of miRNAs [5, 6]. Furthermore, these studies have demonstrated that miRNAs are associated with many important biological processes, such as viral infection [7], immune reaction [8], tumor invasion [9], signal transduction [10], cell proliferation [11], cell growth [12], and cell death [13]. With the development of biotechnology, more and more miRNA-disease associations have been revealed. By studying the expression changes of cancer-associated miRNAs in the early stage of HBV-associated hepatocarcinogenesis, Gao *et al*. found that the deregulation of miRNAs is an early event and accumulates in various steps of HBV-associated hepatocarcinogenesis. At the same time, their results also indicate that miR-145 is a candidate tumor suppressor miRNA, which may play an important role in the development of HCC [14]. Bang *et al*. discovered that miR-23, miR-27 and miR-24 cluster are involved in angiogenesis and endothelial apoptosis during cardiac ischemia and retinal vascular development, and plays an important role in cardiovascular angiogenesis [15]. However, the traditional experimental methods have the disadvantages of long experimental cycle, high cost, small scale and easy to be disturbed by the outside world. Therefore, researchers are committed to finding more efficient computational methods to achieve large-scale and credible predictions of the association among miRNAs and diseases.

Based on the hypothesis that functionally similar miRNAs tend to be associated with diseases with similar phenotypes, many computational methods for predicting miRNA-disease association have been proposed [16–18]. These computational methods can be roughly divided into two categories: similarity-based measures methods and machine learning-based methods [19–21]. The former predicts miRNA-disease association by measuring the association strength between nodes in miRNA and disease network, while the latter applies the machine learning correlation algorithm to this problem [22–24]. Chen *et al*. proposed the RWRMDA method and applied it to the miRNA-miRNA functional similarity network, which starts at a given seed node and randomly simulates the transfer process of the pedestrian from the current node to its neighboring nodes in the network, thus predicting the relationship between miRNA and disease [25]. Liu *et al*. constructed a heterogeneous network by combining data from multiple sources and applied the random walk algorithm to predict miRNA-disease associations. In this method, the functional similarity information of miRNA, semantic similarity information of diseases and miRNA-disease association information are added to the network model, so that it can predict the potential association of new diseases with unknown miRNA related information [26]. Zeng *et al*. proposed a prediction method based on social network analysis, which combines social network analysis with machine learning to predict the relationship between miRNA and disease under the premise of known miRNA-disease association, miRNA-miRNA functional similarity, and disease-disease similarity [27]. Zou *et al*. used a supervised machine learning approach to predict miRNA-disease associations by training the biased SVM classifier with bootstrap aggregating algorithm [28].

**Fig 1:**
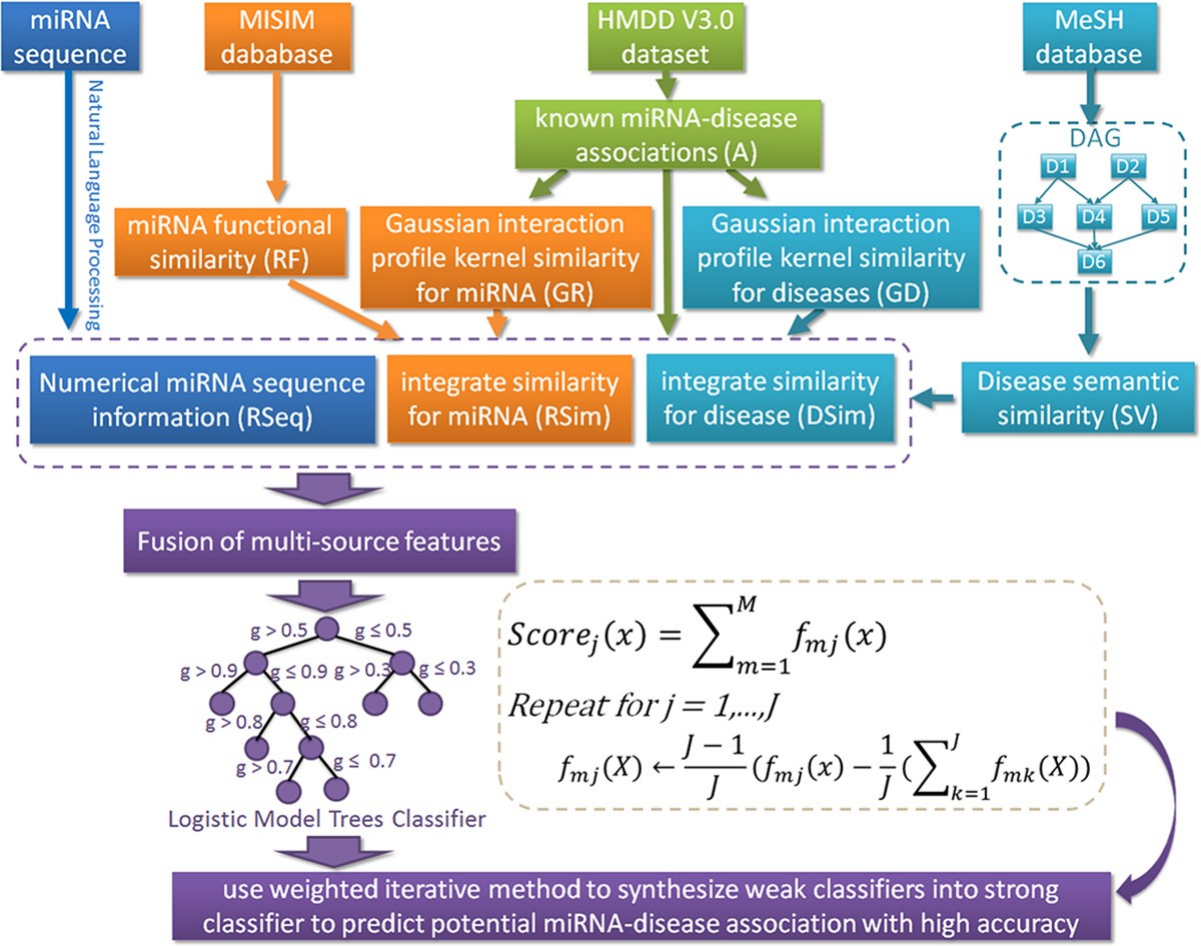
Flowchart of LMTRDA model to predict potential miRNA-disease associations.

In this study, we propose a new computational method of Logistic Model Tree for predicting miRNA-Disease Association (LMTRDA) based on the assumption that functionally similar miRNAs are often associated with phenotypically similar diseases, and vice versa. The LMTRDA combines multiple sources of data information, including miRNA sequence information, miRNA functional similarity information, disease semantic similarity information, and known miRNA-disease association information. In particular, LMTRDA incorporates biological sequence information of miRNAs extracted by natural language processing techniques. Specifically, LMTRDA first respectively calculates the similarity between miRNA and disease according to the miRNA functional similarity network and disease semantic similarity network, and combines them with the Gaussian interaction profile kernel similarity network to obtain the similarity descriptors of miRNA and disease. Secondly, the Natural Language Processing (NLP) technology is used to extract the feature information of the miRNA sequence, and the sequence information and the similarity information of each miRNA-disease pair are combined to form a complete feature descriptor according to the known miRNA and disease association. Finally, the reduced dimension feature descriptors are fed into the Logistic Model Tree (LMT) classifier to predict the associations among miRNAs and diseases. The flowchart of LMTRDA model to predict potential miRNA-disease associations is shown in Fig 1. To evaluate the performance of LMTRDA, the five-fold cross-validation was implemented on the newly released HMDD V3.0 dataset. As a result, LMTRDA obtained 90.51% prediction accuracy with 92.55% sensitivity at the AUC of 90.54%. In comparison with different classifiers and feature descriptors, LMTRDA also achieved good results. Furthermore, we validated the proposed model against three human diseases including Breast Neoplasms, Colon Neoplasms and Lymphoma. Ultimately, most of the top 30 miRNA candidates associated with these three diseases (28 of 30 in Breast Neoplasms, 27 of 30 in Colon Neoplasms, 26 of 30 in Lymphoma) predicted by LMTRDA were confirmed in some representative databases. These experimental results indicated that LMTRDA is well suitable for predicting miRNA-disease association.

## Materials and methods

### Human miRNA–disease association dataset

In the experiment, we validate our model using the HMDD (Human microRNA Disease Database) dataset provided by Li *et al*. [29]. The HMDD dataset provides experiment-supported evidence for human miRNA and disease association, which collects miRNA and disease association data from the evidence of circulating miRNAs, epigenetics, genetics and miRNA-target interactions, and contains detailed and comprehensive annotations. Currently, the latest version of the HMDD dataset is V3.0, which collects 32281 miRNA-disease association entries, including 1102 miRNAs and 850 diseases from 17412 papers. This dataset can be downloaded from the http://www.cuilab.cn/hmdd. When pre-processing the dataset, we removed some of the miRNAs because their information was judged to be unreliable by the public database miRBase. After screening, we chose 32226 miRNA-disease association pairs containing 1057 miRNAs and 850 diseases as positive samples in the experiment. Since HMDD does not provide unrelated miRNA-disease association entries, we randomly selected 32226 miRNA-disease pairs as negative samples from all possible miRNA-disease pairs that have removed the positive samples. In fact, the negative sample set thus constructed may contain positive samples that have not been confirmed by the experiment. However, from a statistical point of view, the proportion of negative samples we selected from all possible samples is only 32226÷(850×1057)≈0.0358, and the number of samples with actually interactions as negative sample sets is very small. Ultimately, the dataset used in our experiment contained 64456 samples, of which positive and negative samples accounted for half. On this basis, we constructed the adjacency matrix *AD* of miRNA and disease, which consists of 850 rows and 1057 columns, corresponding to 850 diseases and 1057 miRNAs, respectively. When disease *d*(*i*) and miRNA *m*(*j*) are verified to be related by the HMDD V3.0 database, the element *AD*(*d*(*i*),*m*(*j*)) of the adjacency matrix *AD* is assigned to 1, otherwise it is assigned to 0. Known human miRNA-disease associations and their names obtatined from HMDD V3.0 database can be seen in S1–S3 Tables.

### Disease semantic similarity

The disease semantic similarity information we use comes from the MeSH database, which can be downloaded from the National Library of Medicine database at https://www.nlm.nih.gov/. The MeSH database gives a rigorous disease classification system of diseases, which provides great help for the study of disease semantic similarity [30]. In the system, the relationship among diseases is described as the Directed Acyclic Graph (DAG), where node represents disease and edge represents their relationship [31]. If the disease *d*(*i*) is related to the disease *d*(*j*), use the edge to connect them, indicating that the child node *d*(*i*) comes from the parent node *d*(*j*). Thus, disease *d*(*i*) can be described as *DAG*_*d*(*i*)_ = (*d*(*i*),*N*_*d*(*i*)_,*E*_*d*(*i*)_), where *N*_*d*(*i*)_ is the ancestor node set of *d*(*i*) including *d*(*i*), and *E*_*d*(*i*)_ is the edge set containing the corresponding edges. We define the contribution of disease *s* in *DAG*_*d*(*i*)_ to the semantic value of disease *d*(*i*) as follows:

$$\left\{ \begin{array}{l} D_{d(i)}(s)=1\;if\;s=d(i)\\ D_{d(i)}(s)=\max\left\{\varepsilon \cdot D_{d(i)}(s^{\prime })|s^{\prime }\in children\;of\;s\right\}\;if\;s\neq d(i) \end{array}\right.$$ (1)

Where *ε* is the semantic contribution factor linking disease *s* and its child disease *s*′. In the DAG of disease *d*(*i*), the contribution value of disease *d*(*i*) to its own semantic value is defined as 1. Therefore, we can get the semantic value *DV*(*d*(*i*)) of disease *d*(*i*), and its formula is as follows:

$$DV(d(i))=\sum_{s\in N_{d(i)}}D_{d(i)}(s)$$ (2)

Here, we assume that diseases sharing more parts of their DAGs will have higher semantic similarity. By considering the relative position of disease *d*(*i*) and disease *d*(*j*) in the MeSH disease DAG, the semantic similarity value *SV*_1_(*d*(*i*),*d*(*j*)) between them can be calculated, and the formula is as follows.

$${SV}_{1}(d(i),d(j))=\frac{\sum_{s\in N_{d(i)}\cap N_{d(j)}}\left(D_{d(i)}(s)+D_{d(j)}(s)\right)}{DV(d(i))+DV(d(j))}$$ (3)

In the *SV*_1_ model, we mainly consider the relationship between the layers of disease in DAG graph, that is, the contribution of different diseases in the same layer to the semantic value is the same. However, we observed that the number of different diseases appearing in the DAGs is different, and the contribution of disease less appearing in the DAGs should be higher than that of disease more appearing in the DAGs. Therefore, in order to distinguish this situation, we introduce the second calculation model [32] of contribution value of disease *s*, the formula is as follows:

$$D_{d(i)}^{\prime }(s)=-log(\frac{num(DAGs(s))}{num(diseases)})$$ (4)

where *num*(*DAGs*(*s*)) indicates the number of DAGs containing disease *s*, and n*um*(*diseases*) indicates the number of all diseases. Thus, the second model of semantic similarity value *SV*_2_(*d*(*i*),*d*(*j*)) of disease *d*(*i*) and disease *d*(*j*) is obtained, and the formula is as follows:

$${SV}_{2}(d(i),d(j))=\frac{\sum_{s\in N_{d(i)}\cap N_{d(j)}}\left(D_{d(i)}^{\prime }(s)+D_{d(j)}^{\prime }(s)\right)}{DV(d(i))+DV(d(j))}$$ (5)

where the value of *DV*(*d*(*i*)) and *DV*(*d*(*j*)) are the same as model 1, which can be calculated using formula 2. The diseases used in disease similarity model 1 and model 2 are from the MeSH database, which accounts for only a part of the diseases we use. Therefore, the remaining disease similarity scores are calculated using Gaussian interaction profile kernel similarity.

### MiRNA functional similarity

Under the hypothesis that functionally similar miRNAs are more likely to be associated with phenotypically similar diseases, Wang *et al*. proposed a functional similarity model to calculate the functional similarity between different miRNAs [31], and placing its functional similarity score matrix at http://www.cuilab.cn/files/images/cuilab/misim.zip. In this article, we download it as the miRNA function similarity information. But similar to the case of the disease similarity model, the miRNAs provided in this matrix contains barely a portion of the miRNAs we use. Therefore, we combine it with Gaussian interaction profile kernel similarity to form a complete miRNA similarity matrix. The constructed miRNA functional similarity score matrix can be seen in S4 Table.

### Gaussian interaction profile kernel similarity

Since the HMDD V3.0 dataset provides a greater number of diseases and miRNAs than the disease and the miRNA similarity models described above, we describe the remaining disease and miRNA similarity information using Gaussian interaction profile kernel similarity [33]. The calculation of Gaussian interaction profile kernel similarity for diseases is based on the hypothesis that similar diseases tend to be functionally similar miRNA, and vice versa. By observing whether disease *d*(*i*) is associated with each of the 1057 miRNAs we have compiled from the HMDD V3.0 dataset, we defined binary vector *V*(*d*(*i*)) to represent the interaction profiles of disease *d*(*i*). Here, the binary vector *V*(*d*(*i*)) is the row vector of the adjacency matrix *AD* in which the disease *d*(*i*) is located. Gaussian interaction profile kernel similarity for diseases *GD*(*d*(*i*),*d*(*j*)) between disease *d*(*i*) and disease *d*(*j*) can be calculated as follows:

$$GD(d(i),d(j))=exp(-\theta_{d}{\left\|V(d(i))-V(d(j))\right\|}^{2})$$ (6)

where *θ*_*d*_ is the width parameter of the function, which can be calculated by normalizing the original parameters. The formula is as follows:

$$\theta_{d}=\frac{1}{m}\sum_{i=1}^{m}{\left\|V(d(i))\right\|}^{2}$$ (7)

where *m* is the number of rows of the adjacency matrix *AD*.

Similarly, Gaussian interaction profile kernel similarity for miRNA *GR*(*r*(*i*),*r*(*j*)) between miRNA *r*(*i*) and miRNA *r*(*j*) can be calculated as follows:

$$GR(r(i),r(j))=exp(-\theta_{r}{\left\|V(r(i))-V(r(j))\right\|}^{2})$$ (8)

$$\theta_{r}=\frac{1}{n}\sum_{i=1}^{n}{\left\|V(r(i))\right\|}^{2}$$ (9)

where the binary vector *V*(*r*(*i*)) is the column vector of the adjacency matrix *AD* in which the miRNA *r*(*i*) is located, *n* is the number of columns of the adjacency matrix *AD*.

### Numerical representation of miRNA sequences

The sequence of miRNA contains abundant information. In order to describe the characteristics of miRNA more comprehensively, we transform them into numerical vectors and fuse them with the above similarity vectors to form the final descriptors. The usual approach to convert miRNA sequences into numerical vectors is to use k-mers [34], which refers to the length of a subsequence of *k*. Given a miRNA sequence of length *l*, the number of possible k-mers is *l*−*k*+1. For example, 6-mers sequence of miRNA can be represented as *AAAAAA*,*AAAAAC*,…,*UUUUUU*. However, this approach does not take into account the difference between the two k-mers because it treats the distance between any two k-mers as equal. But the difference between *AAAAAA* and *UUUUUU* is significantly larger than between *AAAAAA* and *AAAAAC*. Therefore, we introduce natural language processing technology to solve this problem [35–38]. It can not only transform the original high-dimensional data into low-dimensional continuous real-valued vector, but also learn its effective representation from miRNA sequences in an unsupervised manner.

In this study, we use skip-gram in natural language processing's Word2vec algorithm to learn the distributed representation of miRNA for k-mers, which is a shallow two-layer neural network and represents an item by considering its context information from the nearby items. Given a sequence of words *w*_1_,*w*_2_,…,*w*_*n*_, skip-gram uses the co-occurrence information of words in the context window to learn the word representation, and look for the parameter set *θ* to maximize the product of the following conditional probabilities.

$$arg\;\max_{\theta }\prod_{w\in T}\left[\prod_{c\in C(w)}p(c|w;\theta )\right]$$ (10)

where *T* is the text set; *w* is a word; *c* is a word in the context; *C*(*w*) is the set of words contained in the context in which the word *w* appears in the text set *T*; *p* is a conditional probability, which is defined as follows:

$$p(c|w;\theta )=\frac{exp(v_{c}∙v_{w})}{\sum_{c\prime \in C}exp(v_{c}∙v_{w})}$$ (11)

where v_c_ and v_w_ are the column vectors of *c* and *w*, respectively; *C* is the set of words in all contexts, which is equivalent to vocabulary *v*; and parameter *θ* is the specific value of each dimension in *v*_*c*_ and *v*_*w*_. In experiments, we use 6-mers to transform miRNA sequences, which ultimately get 4^6^ = 4096 6-mers. Taking the AAGUCGUACGAU sequence as an example, 6-mers can convert it to {AAGUCG,AGUCGU,GUCGUA,UCGUAC,CGUACG,GUACGA,UACGAU}. After obtaining the 6-mers of all miRNAs in the HMDD V3.0 dataset, we trained the skip-gram word2vec algorithm using all the miRNAs downloaded from the public database miRBase as training sets. In the implementation of the algorithm, we use the following parameters: the minimum number of occurrences of the training words "min_count" is set to 5, the maximum distance of the word vector context "window" is set to 5, the dimension size of the word vector "size" is set to 64, the maximum number of iterations in the stochastic gradient descent method "iter" is set to 10, and the other parameters are set to default values.

### Multi-source feature fusion

In this study, we ultimately used descriptors that fused multiple sources of data including disease similarity, miRNA similarity and miRNA sequence to predict the miRNA-disease association. The advantage is that it can reflect the characteristics of diseases and miRNAs from different perspectives, help to deeply dig out the potential relationship among miRNAs and diseases, and improve the performance of model prediction.

For the similarity of diseases, we construct disease semantic similarity model *SV*_1_, disease semantic similarity model *SV*_2_ and disease Gaussian interaction profile kernel similarity *GD*. The disease similarity matrix *DSim*(*d*(*i*),*d*(*j*)) between disease *d*(*i*) and *d*(*j*) can be obtained by integrating the above disease similarities. The formula is as follows:

$$DSim(d(i),d(j))=\left\{ \begin{array}{l} \frac{{SV}_{1}(d(i),d(j))+{SV}_{2}(d(i),d(j))}{2}\;if\;d(i)\;and\;d(j)\;has\;semantic\;similarity\\ GD(d(i),d(j))\;otherwise \end{array}\right.$$ (12)

For the similarity of miRNA, we combined miRNA functional similarity *RF* and miRNA Gaussian interaction profile kernel similarity *GR* to form miRNA similarity matrix *RSim*. The miRNA similarity matrix *RSim*(*r*(*i*),*r*(*j*)) formula for miRNA *r*(*i*) and miRNA *r*(*j*) is as follows:

$$RSim(r(i),r(j))=\left\{ \begin{array}{l} RF(r(i),r(j))\;if\;d(i)\;and\;d(j)\;has\;functional\;similarity\\ GR(r(i),r(j))\;otherwise \end{array}\right.$$ (13)

For the final feature vector *FV*, we need to integrate the sequence information of miRNA *RSeq*. The feature vector *FV*(*d*(*i*),*r*(*j*)) formed by diseases *d*(*i*) and miRNA *r*(*j*) can be described in the following formula:

FV(d(i),r(j))=[DSim(d(i)),RSim(r(j)),RSeq(r(j))] (14)

where *DSim*(*d*(*i*)) represents the *i* row vector of disease *d*(*i*) in the disease similarity matrix DSim; *RSim*(*r*(*j*)) represents the *j* column vector of miRNA *r*(*j*) in the miRNA similarity matrix *RSim*; *RSeq*(*r*(*j*)) represents the *j* row vector of miRNA *r*(*j*) in the miRNA sequence matrix *RSeq*.

### Logistic model trees classifier

In this study, we use the Logical Model Tree (LMT) as a classifier to predict the associations among miRNAs and diseases. The basic idea of LMT originates from the combination of two complementary classification schemes: linear logistic regression and tree induction [39, 40]. It uses the LogitBoost algorithm to establish the logistic regression function on the node of the tree, and uses the CART algorithm to prune. Specifically, LMT first constructs a basic "weak classifier" based on the existing sample dataset, and calls the "weak classifier" repeatedly. By giving more weight to the wrong samples in each round, it will pay more attention to the samples that are hard to judge. Then, after several rounds of cycles, the "weak classifiers" of each round are combined into the "strong classifier" by weighting method, thereby obtaining a higher precision prediction model. Finally, the tree grown in the training set is pruned using the CART algorithm to obtain the final classification model.

## Results and discussion

### Evaluation criteria

To have a comprehensive assessment of the performance of LMTRDA, we follow common evaluation criteria to evaluate the model, including accuracy (Accu.), sensitivity (Sen.), precision (Prec.) and Matthews Correlation Coefficient (MCC). Their calculation formulas are defined as follows:

$$Accu.=\frac{TP+TN}{TP+TN+FP+FN}$$ (15)

$$Sen.=\frac{TP}{TP+FN}$$ (16)

$$Prec.=\frac{TP}{TP+FP}$$ (17)

$$MCC=\frac{TP\times TN-FP\times FN}{\sqrt{\left(TP+FP)(TP+FN)(TN+FP)(TN+FN\right)}}$$ (18)

where TP, TN, FP, and FN respectively indicate the number of correctly predicted positive samples, correctly predicted negative samples, incorrectly predicted positive samples, and incorrectly predicted negative samples by the model. In addition, the Receiver Operating Characteristic (ROC) curve and the area under the curve (AUC) that can comprehensively reflect the performance of the model are also used in the experiment [41].

### Assessment of prediction ability

To assess the ability of LMTRDA to predict miRNA-disease association, we validated it on HMDD V3.0 dataset using the five-fold cross-validation by LMT classifier. Firstly, we divided all 64452 miRNA-disease pairs into five subsets that were disjoint and roughly equal. Secondly, four of them are selected as training sets to train the LMT classifier, and the remaining one is used as a test set to obtain prediction results. Finally, take turns selecting different subsets as the test set and repeat step 2 until all subsets are treated as test set once and only once. We collected the results of these five experiments and used the mean and standard deviation as the final experimental results.

**Fig 2:**
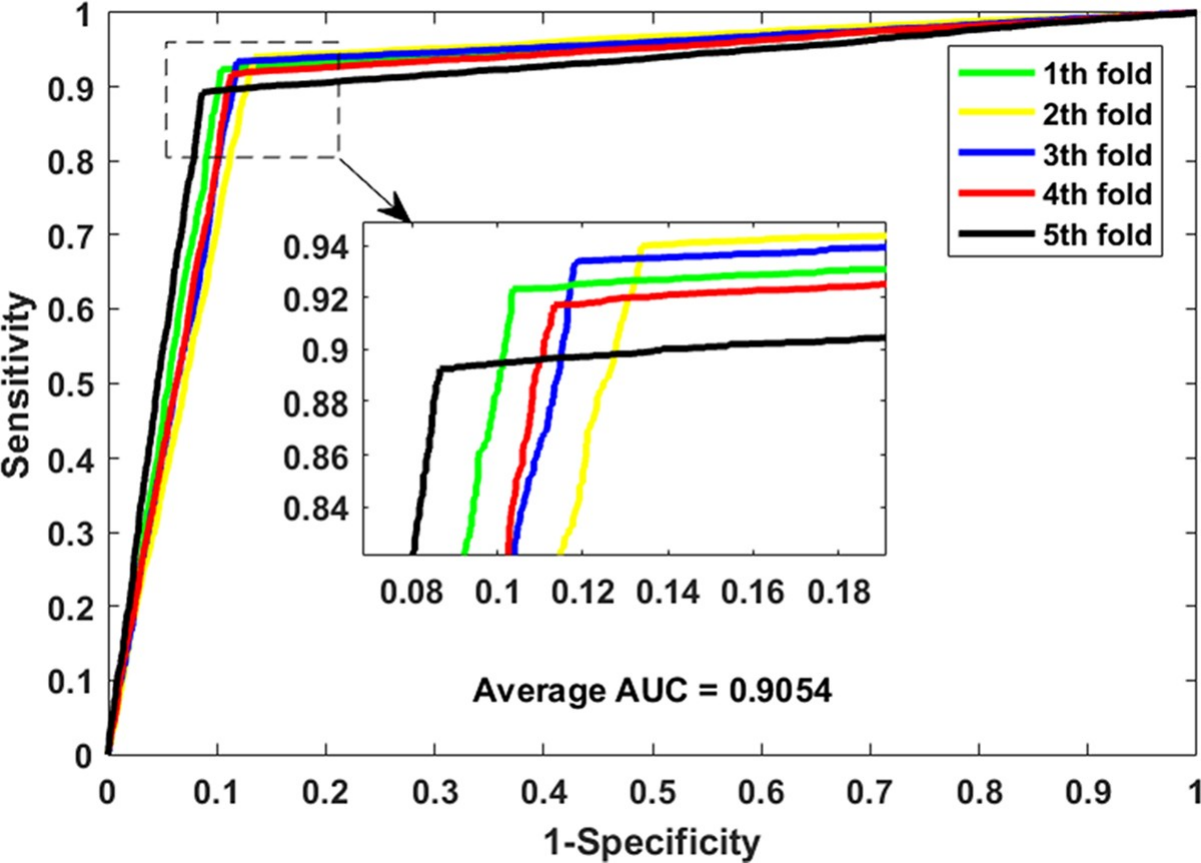
ROC curves performed by LMTRDA on HMDD V3.0 dataset.

**Fig 3:**
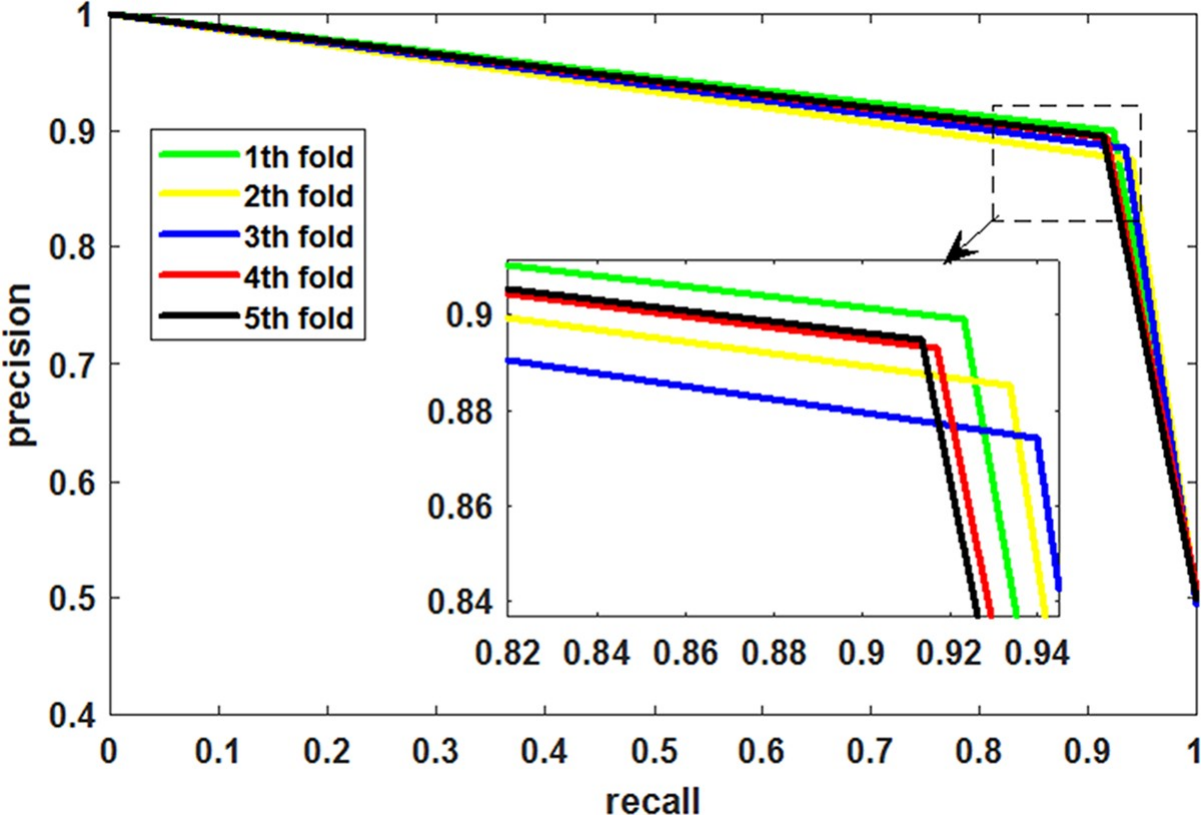
PR curves performed by LMTRDA on HMDD V3.0 dataset.

Table 1 lists the experimental results of the five-fold cross-validation obtained by LMTRDA on the HMDD V3.0 dataset. We can see from the table that LMTRDA has achieved an average prediction accuracy of 90.51%. The accuracy of the five experiments is 90.99%, 90.29%, 90.74%, 90.22% and 90.30% respectively, while the standard deviation is only 0.34%. The LMTRDA model obtained the sensitivity, precision, Matthews correlation coefficient and area under ROC curve are 92.55%, 88.93%, 81.10%, and 90.54%, with standard deviations of 1.11%, 0.98%, 0.67% and 0.33% respectively. The ROC curves and PR curves generated by our proposed method on the HMDD V3.0 dataset are shown in Fig 2 and Fig 3.

**Table 1:** Five-fold cross-validation results performed by LMTRDA on HMDD V3.0 dataset.

Test set	Accu.(%)	Sen.(%)	Prec. (%)	MCC(%)	AUC(%)
1	90.99	92.32	89.92	82.00	91.03
2	90.29	93.98	87.44	80.81	90.51
3	90.74	93.37	88.53	81.60	90.69
4	90.22	91.72	89.31	80.47	90.22
5	90.30	91.35	89.47	80.63	90.27
Average	**90.51±0.34**	**92.55±1.11**	**88.93±0.98**	**81.10±0.67**	**90.54±0.33**

### Comparison among different classifiers

Our proposed LMTRDA model has achieved satisfactory results on HMDD V3.0 dataset using the LMT classifier. In this part of the experiment, we select the state-of-the-art SVM classifier and random forest classifier to compare with it [42]. SVM is a supervised learning algorithm to solve classification problems. It can find the best separated hyperplane in the feature space to maximize the interval between positive and negative samples on the training set, and obtain the global optimization result [43, 44]. Random forest is a classifier with multiple decision trees whose output is determined by the mode number of output categories of decision trees [45, 46]. It can improve the prediction accuracy without significantly improving the amount of computation, so it is widely used in the field of pattern recognition and data mining. When classifying with SVM classifier, we optimized its parameters using grid search method and set the kernel function to radial basis function, *c* = 0.5 and *g* = 0.2. We use radial basis as the kernel function for the SVM classifier, and the optimization results are stored in S5 Table. When classifying with random forest classifier, we also optimized its parameters, setting the maximum depth of the tree to 2, and other parameters to the default values.

Tables 2 and 3 summarize the five-fold cross-validation results performed by SVM and random forest classifier combined with the proposed feature descriptors on the HMDD V3.0 dataset. From Table 2 we can see that the accuracy, sensitivity, precision, MCC, and AUC obtained by the SVM model are 86.09%, 76.14%, 95.05%, 73.65% and 86.10%, and their standard deviations are 0.29%, 0.60%, 0.18%, 0.45% and 0.38%, respectively. It can be seen from Table 3 that the accuracy, sensitivity, precision, MCC, and AUC achieved by random forest model are 89.66%, 88.14%, 90.90%, 79.35% and 89.73% respectively. Their standard deviations are 0.50%, 0.57%, 0.50%, 1.01%, 0.58%, respectively.

**Table 2:** Five-fold cross-validation results performed by SVM classifier combined with the proposed feature descriptors on HMDD V3.0 dataset.

Test set	Accu.(%)	Sen.(%)	Prec. (%)	MCC(%)	AUC(%)
1	86.30	76.56	95.09	74.02	86.13
2	86.46	77.00	94.82	74.21	86.75
3	86.04	75.71	95.06	73.52	85.91
4	85.76	75.65	95.32	73.21	85.84
5	85.88	75.79	94.97	73.28	85.88
Average	**86.09±0.29**	**76.14±0.60**	**95.05±0.18**	**73.65±0.45**	**86.10±0.38**
LMTRDA	**90.51±0.34**	**92.55±1.11**	**88.93±0.98**	**81.10±0.67**	**90.54±0.33**

**Table 3:** Five-fold cross-validation results performed by random forest classifier combined with the proposed feature descriptors on HMDD V3.0 dataset.

Test set	Accu.(%)	Sen.(%)	Prec. (%)	MCC(%)	AUC(%)
1	90.12	88.35	91.60	80.30	90.32
2	89.88	88.69	90.76	79.78	90.14
3	90.02	88.33	91.21	80.06	89.95
4	89.32	88.16	90.55	78.67	89.27
5	88.95	87.18	90.39	77.94	88.97
Average	**89.66±0.50**	**88.14±0.57**	**90.90±0.50**	**79.35±1.01**	**89.73±0.58**
LMTRDA	**90.51±0.34**	**92.55±1.11**	**88.93±0.98**	**81.10±0.67**	**90.54±0.33**

**Fig 4:**
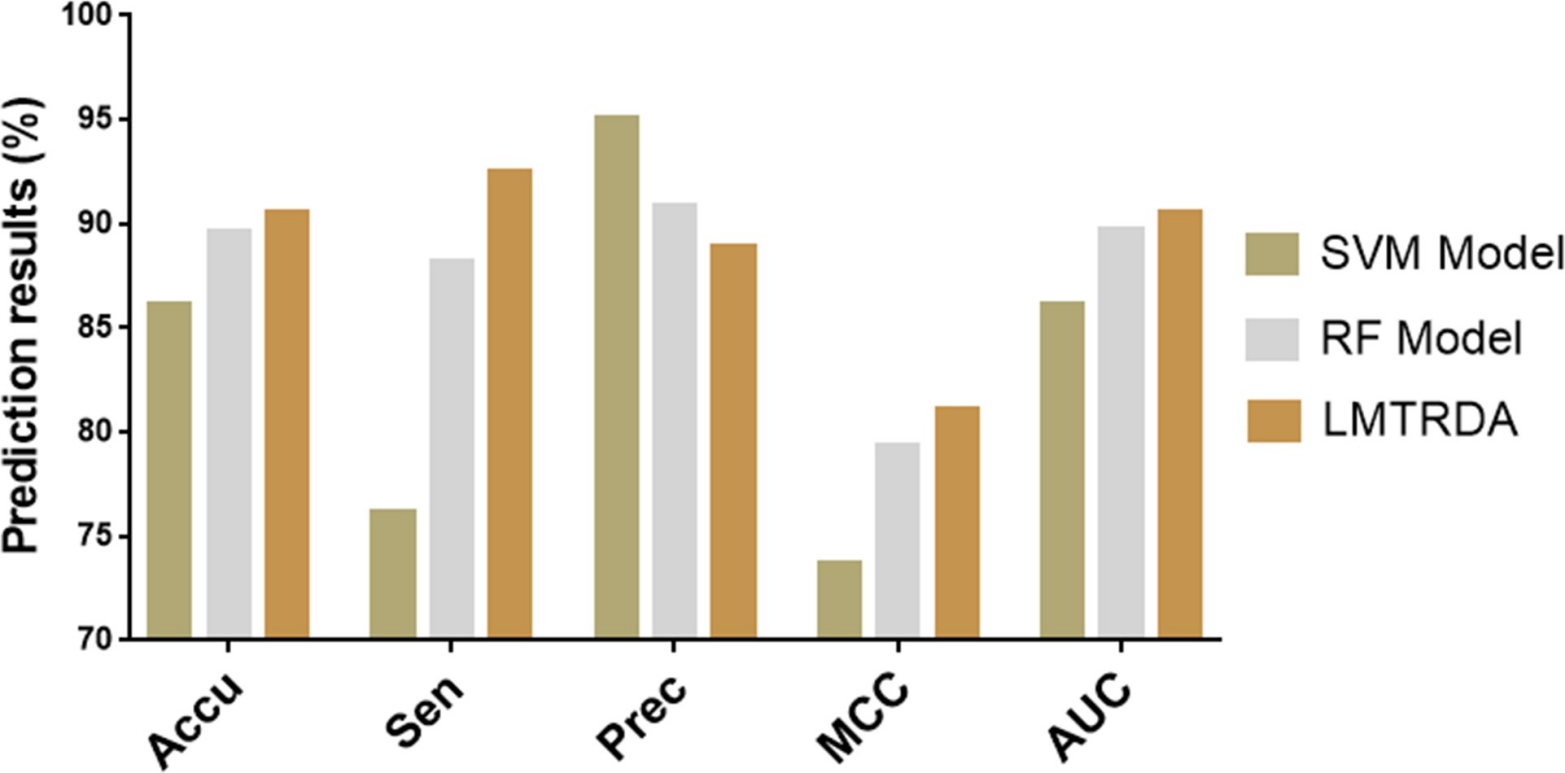
Comparison of results of different classifier models on HMDD V3.0 dataset.

For convenience of comparison, we summarize the experimental results of the three models and present them in the form of the graph. From the Fig 4 we can visually observe that LMTRDA achieves the highest result among the five evaluation criteria of accuracy, sensitivity, MCC, and the third result in terms of precision. This indicates that LMTRDA does not perform as well as the other two models in terms of the precision, which representing the proportion of true positive samples in the positive samples predicted by the prediction model. But overall, the performance of LMTRDA is optimal, especially on the predictive accuracy and the MCC and AUC that represent the overall performance of the model. From Fig 4, we also found that the RF model achieved higher results than that of the SVM model, but generally lower than LMTRDA. This shows that the RF classifier is more suitable for the proposed feature descriptors than the SVM classifier, but the LMT classifier is the most suitable one in this model.

### Comparison among different feature descriptors

To evaluate the ability of our proposed descriptors to represent disease and miRNA feature information, we compare them with different descriptors. Since the descriptor we proposed consists of disease similarity information, miRNA similarity information, and miRNA sequence information, we constructed different descriptors to compare with them in this part of the experiment. That is, the descriptor ‘DescSeq’ consisting only of disease similarity information and miRNA sequence information, and the descriptor ‘DescSim’ consisting only of disease similarity information and miRNA similarity information. Tables 4 and 5 list the five-fold cross-validation results generated by the LMT classifier combined with these two descriptors respectively. It can be seen from the table that the accuracy of the ‘DescSeq’ and ‘DescSim’ descriptors generated on the dataset are 87.51% and 89.43%, the sensitivity are 87.25% and 92.46% and, the precision are 87.71% and 87.23%, the MCC are 75.03% and 79.03%, the AUC are 87.61% and 89.55% and, respectively.

**Table 4:** Five-fold cross-validation results performed by LMT classifier combined with descriptor DescSeq on HMDD V3.0 dataset.

Test set	Accu.(%)	Sen.(%)	Prec. (%)	MCC(%)	AUC(%)
1	88.08	88.83	87.51	76.16	88.13
2	87.55	87.68	87.34	75.10	87.67
3	87.55	87.85	87.09	75.10	87.55
4	87.54	86.43	88.73	75.11	87.73
5	86.84	85.49	87.87	73.70	86.97
Average	**87.51±0.44**	**87.25±1.31**	**87.71±0.64**	**75.03±0.87**	**87.61±0.42**
LMTRDA	**90.51±0.34**	**92.55±1.11**	**88.93±0.98**	**81.10±0.67**	**90.54±0.33**

**Table 5:** Five-fold cross-validation results performed by LMT classifier combined with descriptor DescSim on HMDD V3.0 dataset.

Test set	Accu.(%)	Sen.(%)	Prec. (%)	MCC(%)	AUC(%)
1	90.87	92.31	89.68	81.77	90.92
2	89.95	92.86	87.74	80.03	90.09
3	90.87	92.83	89.43	81.79	90.90
4	87.93	92.25	84.90	76.14	88.15
5	87.55	92.05	84.40	75.42	87.69
Average	**89.43±1.60**	**92.46±0.36**	**87.23±2.48**	**79.03±3.06**	**89.55±1.53**
LMTRDA	**90.51±0.34**	**92.55±1.11**	**88.93±0.98**	**81.10±0.67**	**90.54±0.33**

**Fig 5:**
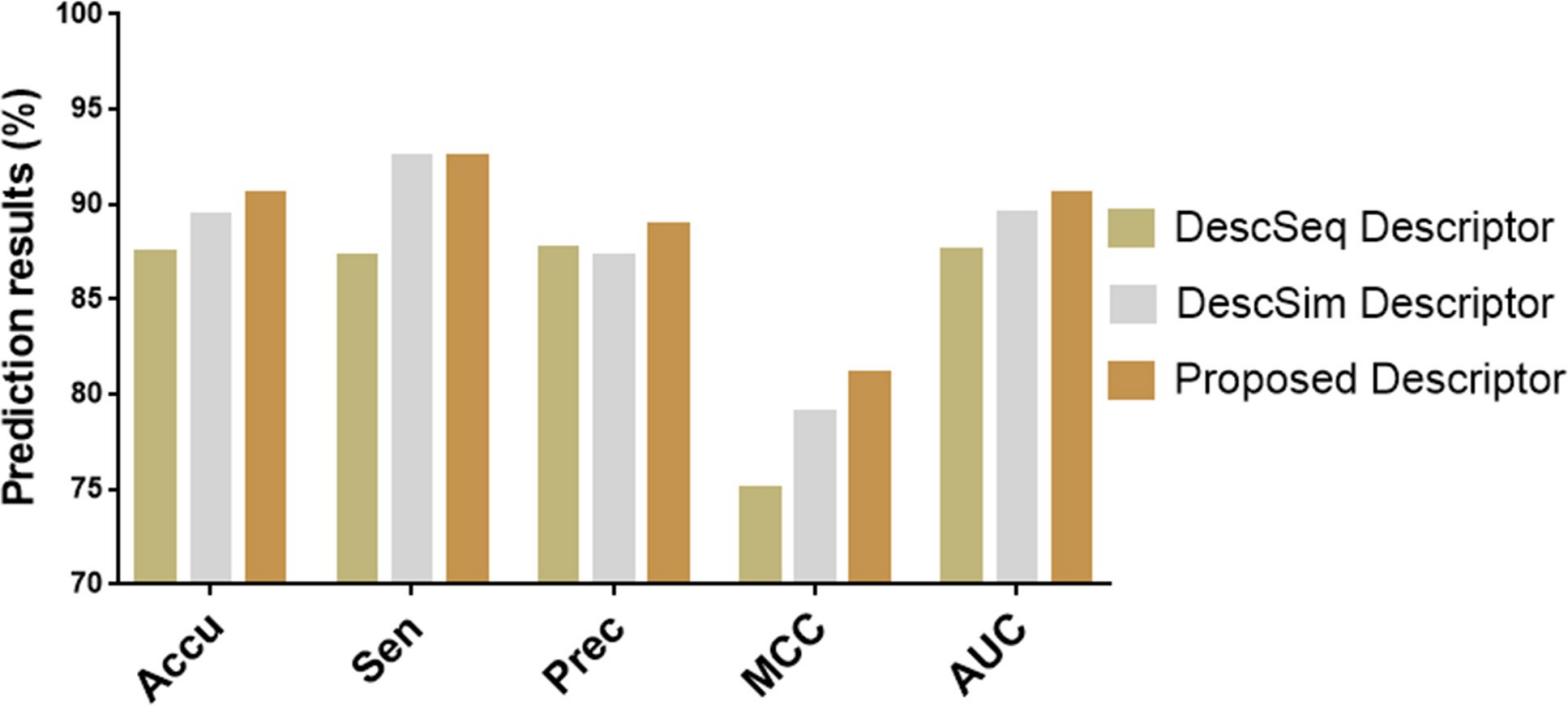
Comparison of results of different descriptor models on HMDD V3.0 dataset.

Fig 5 shows the five-fold cross-validation prediction results of three descriptors combined with LMT classifier on HMDD V3.0 dataset. As can be seen from the Fig 5, our proposed descriptors have achieved the best prediction performance on the evaluation criteria accuracy, sensitivity, precision, MCC, and AUC, respectively. In particular, there is a significant improvement in the Accuracy indicating the average accuracy of the prediction model and the MCC and AUC indicating the overall performance of the prediction model. This suggests that the multi-source information descriptor which combines disease similarity, miRNA similarity and miRNA sequence can describe the miRNA-disease association from different aspects, so as to maximize the deeper meaning of miRNA-disease data hiding.

### Case studies

To further evaluate the performance of LMTRDA, we implemented the case studies on three diseases including Breast Neoplasms, Colon Neoplasms and Lymphoma. In the experiment, we trained the classifier as the training set for all known miRNA-disease pairs in the HMDD V3.0 dataset. The test set is the miRNA-disease pairs consisting of these three diseases and all possible miRNAs. When LMTRDA obtained the predicted results, we took out the 30 miRNAs with the highest scores according to different diseases and verified them in dbDEMC V2.0 and miR2Disease databases [47].

Breast neoplasms are neoplasms that occur in breast tissue, accounting for about two-thirds of breast disease. Malignant breast neoplasms are commonly known as breast cancer, and 99% of them occur in women. The global incidence of breast cancer has been on the rise since the late 1970s, and one in eight women in the United States has breast cancer. At present, breast cancer has become a common neoplasm that threatens women's physical and mental health. A large number of experiments show that many miRNAs are related to breast neoplasms. So we selected breast neoplasms as the first case study and use LMTRDA to predict the miRNAs associate with them. The results are shown in Table 6, 28 out of the top 30 predicted miRNAs are verified in the experimental data provided by the dbDEMC V2.0 and miR2Disease datasets.

**Table 6:** Top 30 miRNAs related to Breast Neoplasms were predicted by LMTRDA based on known miRNA-disease associations in HMDD V3.0 database.

miRNA (prediction score 1–15)	Evidence	miRNA (prediction score 16–30)	Evidence
hsa-mir-520f	dbDEMC V2.0	hsa-mir-211	dbDEMC V2.0
hsa-mir-520e	dbDEMC V2.0	hsa-mir-19b-2	**unconfirmed**
hsa-mir-325	dbDEMC V2.0	hsa-mir-663	dbDEMC V2.0 miR2Disease
hsa-mir-616	dbDEMC V2.0	hsa-mir-362	dbDEMC V2.0
hsa-mir-634	dbDEMC V2.0	hsa-mir-133	dbDEMC V2.0
hsa-mir-637	dbDEMC V2.0	hsa-mir-490	dbDEMC V2.0
hsa-mir-498	dbDEMC V2.0	hsa-mir-483	dbDEMC V2.0
hsa-mir-885	dbDEMC V2.0	hsa-mir-30	dbDEMC V2.0
hsa-mir-181d	dbDEMC V2.0	hsa-mir-186	dbDEMC V2.0
hsa-mir-28	dbDEMC V2.0	hsa-mir-95	dbDEMC V2.0
hsa-mir-216	dbDEMC V2.0	hsa-mir-449b	dbDEMC V2.0
hsa-mir-208b	**unconfirmed**	hsa-mir-330	dbDEMC V2.0
hsa-mir-455	dbDEMC V2.0	hsa-mir-217	dbDEMC V2.0
hsa-mir-382	dbDEMC V2.0	hsa-mir-99b	dbDEMC V2.0 miR2Disease
hsa-mir-520f	dbDEMC V2.0	hsa-mir-365	dbDEMC V2.0

Colon neoplasms are common malignant neoplasms in the gastrointestinal tract, the incidence of which is second only to gastric and esophageal cancer. Lymphoma is a malignant tumor that originates in the lymphoid hematopoietic system. More and more literatures have reported that much miRNAs are closely related to these two diseases. Therefore, we also choose these two diseases as the case study to verify the predictive ability of LMTRDA. Tables 7 and 8 respectively list the top 30 miRNAs with the highest scores associated with the two diseases predicted by LMTRDA. After comparing with the dbDEMC V2.0 and miR2Disease database, 27 out of the top 30 miRNAs in the Colon neoplasms disease predictions can be validated, and 26 out of the top 30 miRNAs can be validated in the Lymphoma disease predictions can be validated.

**Table 7:** Top 30 miRNAs related to Colon neoplasms were predicted by LMTRDA based on known miRNA-disease associations in HMDD V3.0 database.

miRNA (prediction score 1–15)	Evidence	miRNA (prediction score 16–30)	Evidence
hsa-mir-526b	dbDEMC V2.0	hsa-mir-198	dbDEMC V2.0
hsa-mir-520g	dbDEMC V2.0	hsa-mir-181d	dbDEMC V2.0
hsa-mir-520f	dbDEMC V2.0	hsa-mir-181c	dbDEMC V2.0
hsa-mir-520e	dbDEMC V2.0	hsa-mir-181b-2	dbDEMC V2.0
hsa-mir-325	dbDEMC V2.0	hsa-mir-181b-1	dbDEMC V2.0 miR2Disease
hsa-mir-302f	**unconfirmed**	hsa-mir-122	dbDEMC V2.0
hsa-mir-616	dbDEMC V2.0	hsa-mir-370	dbDEMC V2.0
hsa-mir-634	dbDEMC V2.0	hsa-mir-302c	dbDEMC V2.0
hsa-mir-637	dbDEMC V2.0	hsa-mir-28	dbDEMC V2.0
hsa-mir-492	**unconfirmed**	hsa-mir-26a-2	dbDEMC V2.0 miR2Disease
hsa-mir-520c	**unconfirmed**	hsa-mir-26a-1	dbDEMC V2.0 miR2Disease
hsa-mir-520b	dbDEMC V2.0	hsa-mir-216	dbDEMC V2.0
hsa-mir-885	dbDEMC V2.0	hsa-mir-208b	dbDEMC V2.0
hsa-mir-34b	dbDEMC V2.0	hsa-mir-182	dbDEMC V2.0 miR2Disease
hsa-mir-340	dbDEMC V2.0	hsa-mir-103a-2	dbDEMC V2.0

**Table 8:** Top 30 miRNAs related to Lymphoma were predicted by LMTRDA based on known miRNA-disease associations in HMDD V3.0 database.

miRNA (prediction score 1–15)	Evidence	miRNA (prediction score 16–30)	Evidence
hsa-mir-526b	dbDEMC V2.0	hsa-mir-30c-1	dbDEMC V2.0
hsa-mir-520g	dbDEMC V2.0	hsa-mir-198	dbDEMC V2.0
hsa-mir-520f	dbDEMC V2.0	hsa-mir-181d	dbDEMC V2.0
hsa-mir-520e	dbDEMC V2.0	hsa-mir-181b-2	dbDEMC V2.0
hsa-mir-325	dbDEMC V2.0	hsa-mir-506	**unconfirmed**
hsa-mir-302f	**unconfirmed**	hsa-mir-370	dbDEMC V2.0
hsa-mir-616	dbDEMC V2.0	hsa-mir-30a	dbDEMC V2.0 miR2Disease
hsa-mir-634	dbDEMC V2.0	hsa-mir-302c	dbDEMC V2.0
hsa-mir-637	dbDEMC V2.0	hsa-mir-302b	dbDEMC V2.0
hsa-mir-492	dbDEMC V2.0	hsa-mir-216	dbDEMC V2.0
hsa-mir-520b	dbDEMC V2.0	hsa-mir-208b	dbDEMC V2.0
hsa-mir-498	dbDEMC V2.0	hsa-mir-103a-2	**unconfirmed**
hsa-mir-885	dbDEMC V2.0	hsa-mir-103a-1	**unconfirmed**
hsa-mir-340	dbDEMC V2.0	hsa-mir-1	dbDEMC V2.0
hsa-mir-30c-2	dbDEMC V2.0	hsa-mir-499	dbDEMC V2.0

## Conclusion

In this study, we present a novel computational method LMTRDA for predicting miRNA-disease association base on fused multi-source data. An interesting aspect of LMTRDA is the use of natural language processing techniques to transform miRNA sequences into numerical vectors and merge them with miRNA functional similarity, disease semantic similarity, and known miRNA-disease association information to form feature descriptors. Cross-validation experiment results on HMDD V3.0 dataset demonstrated that this model can effectively predict the potential association among miRNAs and diseases. In comparison with different classifier and feature descriptor models, LMTRDA exhibits good performance. In addition, we validated it in human diseases including Breast Neoplasms, Breast Neoplasms and Lymphoma, and LMTRDA also achieved excellent results. These results indicated that LMTRDA is a reliable model for predicting miRNA-disease association. In future research, we will continue to study how to better apply natural language processing techniques to biological sequence data in anticipation of better performance of predictive mod.

## Supporting information

S1 Table Known human miRNA-disease associations obtained from HMDD V3.0 database. (XLSX)

S2 Table Names of 1057 miRNAs involved in known human miRNA-disease associations obtained from HMDD V3.0 database. (XLSX)

S3 Table Names of 850 diseases involved in known human miRNA-disease associations obtained from HMDD V3.0 database. (XLSX)

S4 Table The constructed miRNA functional similarity score matrix. (XLSX)

S5 Table The five-fold cross-validation results using different kernel functions of SVM on HMDD v3.0 dataset. (DOCX)

S1 Fig The disease DAGs of liver neoplasms and pancreatic neoplasms. (TIF)

S2 Fig Procedure of 10-fold cross-validation and 5-fold cross-validation. (TIF)

S3 Fig The process of splitting miRNA sequence into smaller k-mers (2-mers, 3-mers and 4-mers in this case). (TIF)

S4 Fig The schematic diagram of the transformation of miRNA sequence by 6-mers method. (TIF)

S5 Fig Comparison of ROC curves generated by different classifiers on HMDD v3.0 datasets. (TIF)
