# Supplementary material for: LMTRDA: Using logistic model tree to predict MiRNA-disease associations by fusing multi-source information of sequences and similarities
Source: PLoS Comput Biol. 2019 Mar 27;15(3):e1006865. doi: 10.1371/journal.pcbi.1006865 (PMC6464243; doi:10.1371/journal.pcbi.1006865)
Supplement: S5 Table — (DOCX) [file pcbi.1006865.s005.docx]

Supplementary Table 5. The five-fold cross-validation results using different kernel functions of SVM on HMDD v3.0 dataset.

| **Test set** | **Kernel** | **Accu.(%)** | **Sen.(%)** | **Prec.(%)** | **MCC(%)** |
| --- | --- | --- | --- | --- | --- |
| 1 | polynomial | 85.95 | **88.78** | 82.30 | 72.09 |
|  | radial basis | **86.30** | 76.56 | **95.09** | **74.02** |
| 2 | polynomial | 86.29 | **82.90** | 90.95 | 72.95 |
|  | radial basis | **86.46** | 77.00 | **94.82** | **74.21** |
| 3 | polynomial | 81.80 | **76.92** | 90.31 | 64.62 |
|  | radial basis | **86.04** | 75.71 | **95.06** | **73.52** |
| 4 | polynomial | **86.65** | **84.48** | 90.28 | **73.44** |
|  | radial basis | 85.76 | 75.65 | **95.32** | 73.21 |
| **5** | polynomial | 85.70 | **83.38** | 89.18 | 71.57 |
|  | radial basis | **85.88** | 75.79 | **94.97** | **73.28** |
