# Supplementary figures and images for: LMTRDA: Using logistic model tree to predict MiRNA-disease associations by fusing multi-source information of sequences and similarities
Source: PLoS Comput Biol. 2019 Mar 27;15(3):e1006865. doi: 10.1371/journal.pcbi.1006865 (PMC6464243; doi:10.1371/journal.pcbi.1006865)

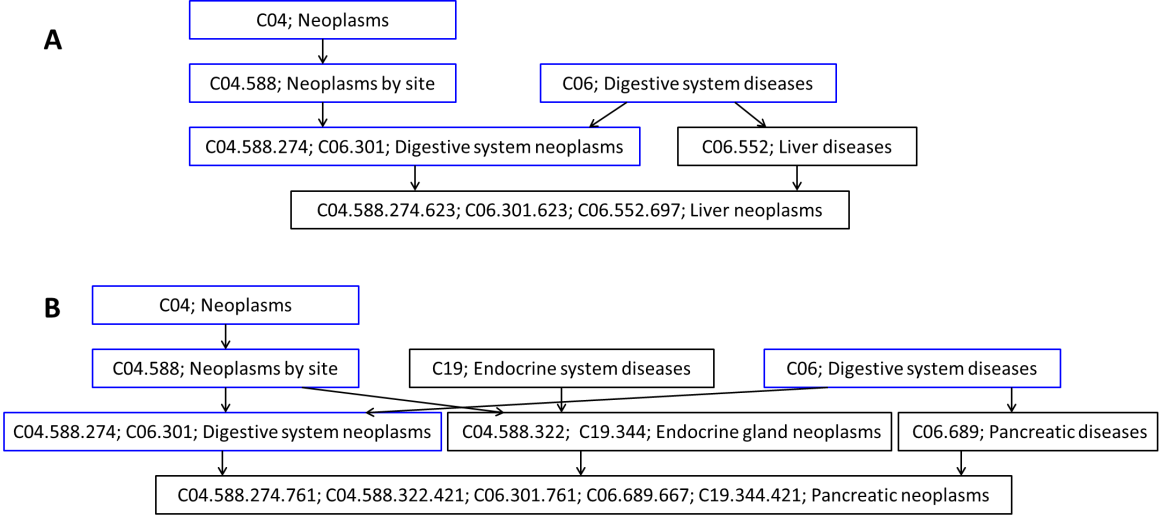

Supplement: S1 Fig — (TIF) [file pcbi.1006865.s006.tif]

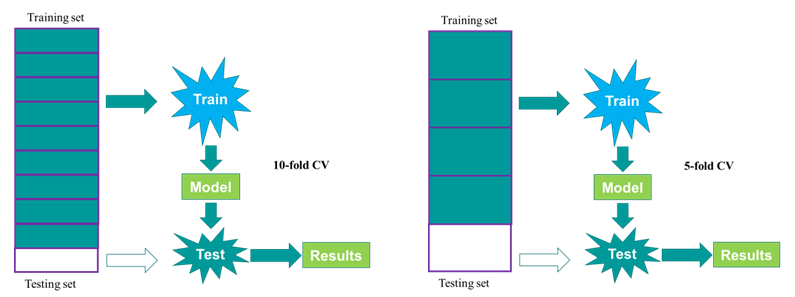

Supplement: S2 Fig — (TIF) [file pcbi.1006865.s007.tif]

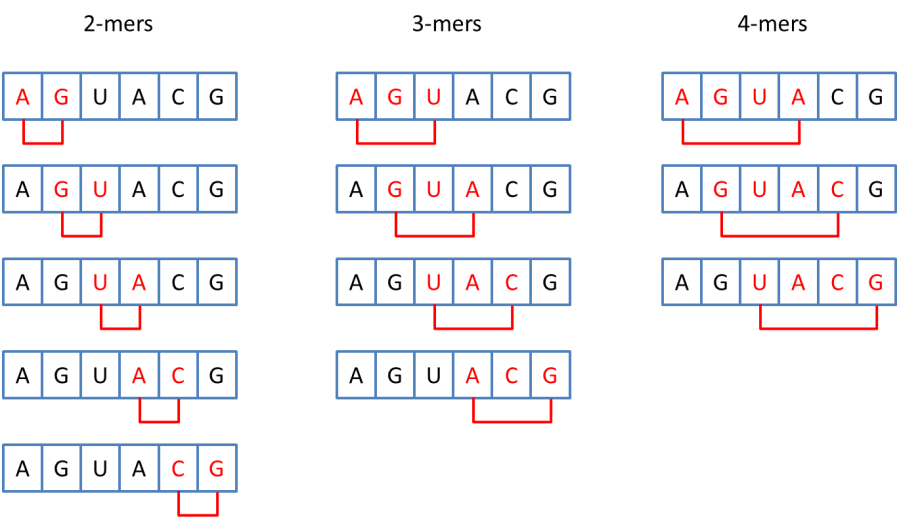

Supplement: S3 Fig — (TIF) [file pcbi.1006865.s008.tif]

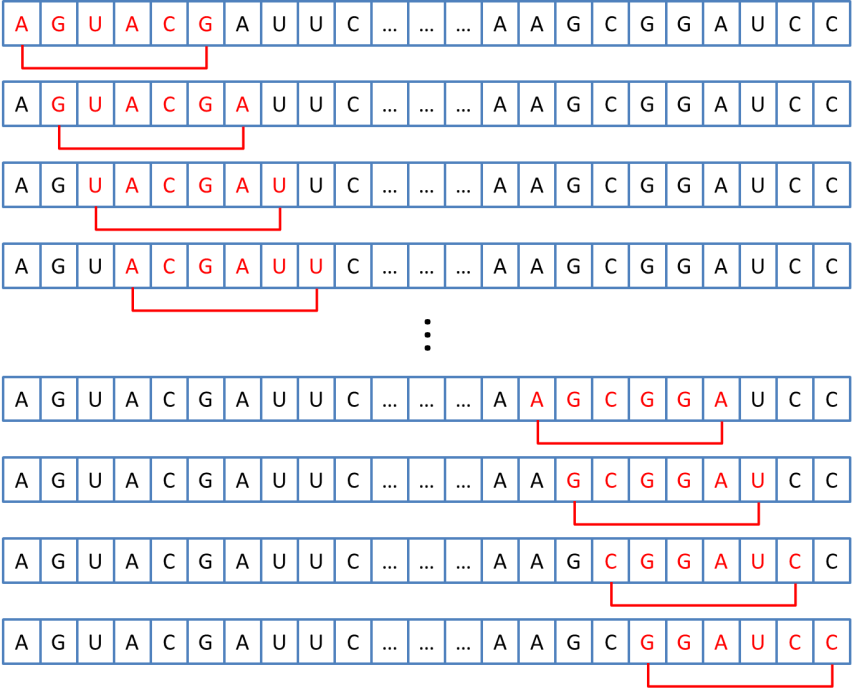

Supplement: S4 Fig — (TIF) [file pcbi.1006865.s009.tif]

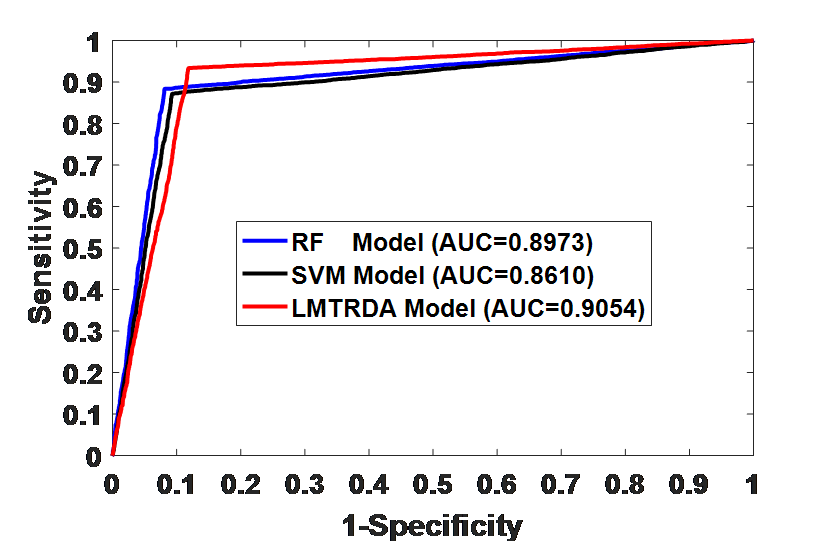

Supplement: S5 Fig — (TIF) [file pcbi.1006865.s010.tif]
